# Substrate-induced Nuclear Export and Peripheral Compartmentalization of Hepatic Glucokinase Correlates with Glycogen Deposition

**DOI:** 10.1155/EDR.2001.173

**Published:** 2001

**Authors:** Thomas L. Jetton, Masa Shiota, Susan M. Knobel, David W. Piston, Alan D. Cherrington, Mark A. Magnuson

**Affiliations:** 1 Department of Molecular Physiology and Biophysics Vanderbilt University School of Medicine Nashville TN 37232-0615 USA; 2 C-349 Given Building Department of Medicine Division of Endocrinology University of Vermont Burlington VT 05405 USA

## Abstract

Hepatic glucokinase (GK) is acutely regulated by binding to its nuclear-anchored regulatory protein (GKRP). Although GK release by GKRP is tightly coupled to the rate of glycogen synthesis, the nature of this association is obscure. To gain insight into this coupling mechanism under physiological stimulating conditions in primary rat hepatocytes, we analyzed the subcellular distribution of GK and GKRP with immunofluorescence, and glycogen deposition with glycogen cytochemical fluorescence, using confocal microscopyand quantitative image analysis. Following stimulation, a fraction of the GK signal translocated from the nucleus to the cytoplasm. The reduction in the nuclear to cytoplasmic ratio of GK, an index of nuclear export, correlated with a >50% increase in glycogen cytochemical fluorescence over a 60min stimulation period. Furthermore, glycogen accumulation was initially deposited in a peripheral pattern in hepatocytes similar to that of GK. These data suggest that a compartmentalization exists of both active GK and the initial sites of glycogen deposition at the hepatocyte surface.


Int. Jnl. Experimental Diab. Res., Vol. 2, pp. 173–186
Reprints available directly from the publisher
Photocopying permitted by license only

© 2001 OPA (Overseas Publishers Association) N.V.
Published by license under
the Harwood Academic Publishers imprint,
part of Gordon and Breach Publishing,
a member of the Taylor & Francis Group.
Printed in the U.S.A.

### Substrate-induced Nuclear Export and Peripheral Compartmentalization of Hepatic Glucokinase Correlates with Glycogen Deposition

THOMAS L. JETTON*, MASA SHIOTA, SUSAN M. KNOBEL, DAVID W. PISTON, ALAN D. CHERRINGTON and MARK A. MAGNUSON

*Department of Molecular Physiology and Biophysics, Vanderbilt University School of Medicine, Nashville, TN 37232-0615*

*(Received 16 April 2001; Revised 11 July 2001; In final form 20 July 2001)*

**Hepatic glucokinase (GK) is acutely regulated by binding to its nuclear-anchored regulatory protein (GKRP). Although GK release by GKRP is tightly coupled to the rate of glycogen synthesis, the nature of this association is obscure. To gain insight into this coupling mechanism under physiological stimulating conditions in primary rat hepatocytes, we analyzed the subcellular distribution of GK and GKRP with immunofluorescence, and glycogen deposition with glycogen cytochemical fluorescence, using confocal microscopy and quantitative image analysis. Following stimulation, a fraction of the GK signal translocated from the nucleus to the cytoplasm. The reduction in the nuclear to cytoplasmic ratio of GK, an index of nuclear export, correlated with a >50% increase in glycogen cytochemical fluorescence over a 60 min stimulation period. Furthermore, glycogen accumulation was initially deposited in a peripheral pattern in hepatocytes similar to that of GK. These data suggest that a compartmentalization exists of both active GK and the initial sites of glycogen deposition at the hepatocyte surface.**

*Keywords*: Glucokinase; Glucokinase regulatory protein; Glycogen synthesis; Confocal microscopy; Immunofluorescence; Nuclear translocation

*Abbreviations*: Glucokinase (GK); Glucokinase regulatory protein (GKRP); Glucose-6-phosphate (G6P); Nuclear to cytoplasmic ratio [N:C]; Periodic Acid-Schiff reaction (PAS)

### INTRODUCTION

Glucokinase (GK) plays a critical role in glucose homeostasis by catalyzing the phosphorylation of glucose in both hepatocytes and pancreatic $\beta$ cells. The short-term regulation of hepatic GK is governed by dynamic and reversible interactions with the GK regulatory protein (GKRP), a nuclear protein.[1] The association/dissociation of GK with GKRP is determined by the exposure to certain hexose phosphates and other substrates such that GKRP functions as a metabolic sensor (see review by[2]). Under basal glucose conditions, GK is predominantly bound to GKRP[3] within the hepatocyte nucleus,[4] where it resides in an inactive state until it is released and translocates to the cytoplasm.[2] Dietary intake influences the subcellular translocation of GK as well as the expression of both GK and GKRP mRNAs.[5,6,7] Impaired GK translocation, such as that observed in animal models of type 2 diabetes,[8] may contribute to altered hepatic glucose metabolism.

There are recent reports that the interplay between hepatic GK and GKRP impacts the acute regulation of glycogen synthesis.[9,10] It has also

*Address for correspondence: C-349 Given Building, Department of Medicine, Division of Endocrinology, University of Vermont, Burlington, VT 05405. Tel.: 802-656-2616, Fax: 802-656-8031, e-mail: tjetton@zoo.uvm.edu been observed that both GK[11] and GKRP-deficient[12] mice exhibit impaired glycogen production. Furthermore, a recent study by de la Iglesia *et al.*[13] demonstrated a very high control coefficient of the GK-GKRP protein ratio on glycogen synthesis. However, there are few details on the subcellular compartmentalization of GK and GKRP under conditions that favor glycogen deposition. Nonetheless, glycogen synthase, the rate-determining enzyme in glycogen synthesis[14] has been found to undergo a glucose-stimulated translocation to the hepatocyte surface coincident with peripheral glycogen deposition.[15]

Despite the well-accepted view that GK shuttles from the nucleus upon substrate stimulation, there are disagreements as to the subcellular disposition of GKRP under these conditions. For example, previous reports have suggested that both GK and GKRP translocate from the nucleus to the cytoplasm upon stimulation with supraphysiological glucose concentrations,[1,16] but other studies using cell-free systems[3] and cultured primary hepatocytes[4] indicate that GKRP remains associated with the nuclear matrix after GK is released. Notwithstanding, we have recently shown that GKRP may not only function in the nuclear sequestration of GK, but may also control nuclear entry of GK.[17] To determine whether the translocation of hepatic GK is spatially coupled with glycogen deposition, and to gain a better understanding of the subcellular dynamics of hepatic GK with GKRP, we investigated the interrelationships between GK, GKRP and glycogen deposition at physiological glucose concentrations in primary hepatocyte cultures. We have devised a simple, reliable method to quantitatively analyze hepatic glycogen *in situ* and compare those patterns with GK immunoreactivity at the subcellular level.

### MATERIALS AND METHODS

### Hepatocyte Isolation and Culture

Primary hepatocytes were isolated from overnight-fasted Sprague-Dawley rats (~300g) using the recirculating collagenase-perfusion method of Shiota *et al.*[18] Hepatocytes were washed in Krebs-bicarbonate buffer (115mmol/l NaCl, 5.9mmol/l KCl, 1.2mmol/l $MgCl_2$, 1.2mmol/l $NaH_2PO_4$, 1.2mmol/l $Na_2SO_4$, and 25mmol/l $NaHCO_3$), and suspended in Dulbecco's modified Eagle's medium (DMEM) with 10% fetal calf serum supplemented with 100U/ml penicillin and 100μg/ml streptomycin. Cell viability was determined by Trypan blue staining and was >95%. Hepatocytes were then plated onto collagen type IV (Gibco)-coated 35mm microwell dishes (Mat-Tek Corp., Ashland, MA) at a density of ~$2.5 \times 10^5$/dish. After 3-4h at 37°C in 5% $CO_2$/95% air, the attached cells were briefly rinsed and incubated with fresh media (DMEM with 5.5mmol glucose with antibiotics and 1μg/ml insulin) and incubated overnight. The media was changed the next morning with DMEM with 5.5mmol/l glucose (basal medium), and the hepatocytes were incubated for an additional 2h before substrate stimulation.

### Isolated Liver Perfusion

Overnight-fasted rats (~300g), were anesthesized and subjected to an isolated, non-recirculating liver perfusion as described above in order to compare subcellular glycogen deposition patterns in isolated hepatocytes with those of intact liver.[18] The principles of animal laboratory care were strictly followed according to the guidelines of both the NIH and Vanderbilt's Animal Care Committee. Livers were equilibrated for 60min with oxygenated (95% $O_2$ + 5% $CO_2$) Krebs-bicarbonate buffer then subjected for 30min to either 5.5mmol/l glucose, 20mmol/l glucose, or 5.5mmol/l glucose + 0.2mmol/l fructose (n = 3 livers for each condition). After the final 30min perfusion, the liver samples were immersion fixed in 4.0% paraformaldehyde/0.1mol/l PBS for 3h at 4°C, and routinely processed for paraffin embedding. Five micron sections were subjected to the "PAS" staining procedure (see below).

### Substrate-stimulation

Primary cultured hepatocytes were incubated for either 30 or 60min in pre-warmed basal medium (DMEM with 5.5mmol/l glucose), basal medium supplemented with 0.2mmol/l fructose, DMEM with 10mmol/l glucose, or DMEM with 10mmol/l glucose and 0.2mmol/l fructose. Following the stimulation period, the hepatocytes were briefly washed in PBS (10mmol/l phosphate-buffered saline, pH 7.6) and fixed in 4.0% paraformaldehyde/PBS for 20 min at room temperature.

### Immunocytochemistry of Cultured Hepatocytes

Following fixation, the hepatocytes were washed three times in PBS for 10min, then permeabilized in PBS with 0.1% Triton X-100 for 20min. The cells were then blocked in 5% normal donkey serum and 1.0% BSA/phosphate-buffered saline (PBS) for 1h. The hepatocytes were then incubated for 12h in a 1:1000 dilution of sheep anti-GST-GK serum[19] for detection of GK, a 1:1000 dilution of rabbit anti-rat GKRP serum[20] or an equivalent mixture of the two for double-labelling immunofluorescence. Following 3 × 15min washes in PBS with 0.1% Triton X-100, the secondary antibodies were applied for 1h at room temperature. For quantitative analysis of GK and RP, the secondary antibodies consisted of either "ML"-grade (Jackson Immunoresearch, West Grove, PA) donkey anti-sheep IgG-CY3 or donkey anti-rabbit IgG-CY3, respectively (both diluted 1:1000 in PBS with 1.0% BSA and 0.1% Triton X-100). For double-labelling immunofluorescence, the secondary antibodies consisted of donkey anti-sheep-CY3 (1:1000) and donkey anti-rabbit CY5 (1:500). Control experiments for GK and RP antiserum specificity consisted of incubations with equivalently diluted pre-immune serum. Controls for secondary antibody specificity were as previously detailed.[21] After washing, the cells were mounted in Aqua-PolyMount (Polysciences, Warrington, PA).

### Western Blotting

To confirm antibody specificity in the recognition of GK and GKRP in hepatocyte samples, immunoblot analysis of whole-cell homogenates of freshly-isolated hepatocytes was performed as follows. Approximately 0.25ml of the pelleted hepatocytes were added to 1ml of the homogenization buffer (50mmol/l triethanolamine-HCl, 100mmol/l KCl, 1mmol/l DTT, 5% glycerol, 5% polyethylene glycol, 1mmol/l EDTA, 1mmol/l EGTA, 1mmol/l PMSF, 1μg/ml Pepstatin A, 1μg/ml leupeptin, 0.02% $NaN_3$, 0.5% Triton X-100 at pH·7.3), homogenized with a Polytron Tissuemizer, then centrifuged at 10,000×g for 10min. 10μg of protein extract from the supernatant was added to SDS sample buffer and resolved by 12% SDS-PAGE. Following electrophoretic transfer to PVDF membranes, the blot was blocked in 5% non-fat dry milk and 0.1% Tween 20 in 20mmol/l Tris buffered saline then incubated with anti-GST-GK antiserum diluted 1:5000 in blocking buffer at 4°C overnight. Bound antibody was detected using "ML"-grade horseradish-peroxidase-conjugated donkey anti-sheep IgG (Jackson Immunoresearch, 1:10,000 in same buffer) and an enhanced chemiluminescence method as detailed previously.[22] The same blot was washed and reprobed with rabbit anti-GKRP serum diluted 1:5000, then incubated in "ML"-grade horseradish-peroxidase-conjugated donkey anti-rabbit IgG and developed by chemiluminescence detection.

### Conventional Periodic Acid-Schiff (PAS) Histochemical Staining for Glycogen

For the conventional PAS procedure, paraffin sections of paraformaldehyde-fixed, routinely-processed liver tissue from perfusion experiments were hydrated to water and oxidized for 10min in 0.5%(w/v) periodic acid, washed in DW, then stained for 10min in Schiff's reagent (Fisher Scientific, Pittsburgh, PA). Following rinsing in 9.5%(w/v) sodium metabisulfite, sections were dehydrated and mounted in Permount (Fisher). Transmitted light images were recorded on a Leitz Laborlux S photomicroscope using Ektachrome 160 slide film.

### Fluorescent PAS Staining of Hepatocytes

A modified PAS technique was devised for the fluorescence detection of glycogen in cultured hepatocytes. This was accomplished by a 2min periodic acid (0.5%w/v) oxidation step followed by a 1min Schiff's reagent staining (freshly filtered) to yield a moderate to strong fluorescent signal under appropriate laser excitation (see below) which was not detectable *via* transmitted light microscopy (*i.e.*, typical magenta Schiff's staining). Control experiments included omission of either the oxidation or Schiff's-staining step and resulted in no detectable cytoplasmic fluorescence.

### Semi-quantitative Confocal Microscopy

Parallel dishes (in duplicate) of similarly-treated hepatocytes were fixed and stained for analysis of GK and RP immunoreactivity and for glycogen by PAS fluorescence detection. Quantitation was performed by laser-scanning confocal microscopy using a Zeiss LSM 410 (Vanderbilt Cell Imaging Resource) to record GK- and GKRP-CY3, and PAS-generated fluorescence (using separate samples) using the 543nm excitation line of a HeNe laser. For each field, the microscope was focused to maximize the number of hepatocytes optically-sectioned through the middle of the nucleus. All confocal imaging parameters were identical for each imaged field and a minimum of 4 fields of $200 \times 200\,\mu m$ were captured for each variable thereby yielding ~25–92 cells for subcellular analyses for each sample and each stimulation condition. Grayscale images ($512 \times 512$ pixels) were transferred to a Power Macintosh 8500 running NIH Image (v 1.59) for image analysis.

### Image Analysis

Image files for subcellular analyses were printed to identify and number individual hepatocytes suitable for quantitation. For each cell studied, a 19 pixel diameter circle (349 pixels total), which was approximately the area of an average hepatocyte nucleus, was used to measure mean pixel intensities (range = 0–255 grayscale levels). Only cells with their mid-nuclear region in focus were counted. For GK and RP-stained cells, an equivalent area of cytoplasm was sampled between the nucleus and cell surface thus allowing comparisons of the changes in nuclear intensity to changes in the cytoplasmic intensity (an index expressed as the nuclear to cytoplasmic [N:C] ratio). Due to the limitations in volume sampling in a single optical section, and the fact that potential changes in nuclear GK signal cannot be fully compensated for by a reciprocal change in cytoplasmic intensity, we found this method of nuclear-cytoplasmic sampling to yield reproducible N:C ratio values and was more time-efficient when compared to cell-outline methods. Fluorescent PAS-stained samples were imaged under equivalent conditions and subjected to cytoplasmic intensity analysis using the same sampling strategy as in the GK/RP-stained samples. The lightly-fluorescent, PAS-reactive Golgi regions were avoided in these analyses to prevent overestimation of glycogen signal. Field background fluorescence values were subtracted from intensity values for each cell. Thus, changes in the subcellular distribution of GK and GKRP were expressed as corrected mean N:C pixel intensity ratios, whereas changes in the levels of PAS-reactive glycogen were expressed as corrected mean cytoplasmic intensities. Data presented in Figure 3 resulted from cultured hepatocyte experiments performed from a single isolation, but are representative of the trends observed in GK/GKRP translocation and glycogen deposition experiments under identical conditions from other isolations. The student's t-test in Microsoft Excel was used for probability ($p$) determinations whereby values of $<0.05$ were deemed significant.

### XZ Scanning and Image Formating

For determination of the subcellular localization of GK immunoreactivity and glycogen in hepatocytes under basal and stimulated conditions, confocal scans in the XZ (depth) plane were obtained of representative fields in the XY dimension using the Zeiss LSM software. Densitometric scans of the XY images in the same plane as the XZ images were obtained in NIH Image to allow a graphical comparison of pixel density with the actual XZ scan. All images were converted to an Adobe Photoshop 4.0 format then transferred to a Silicon Graphics Indigo workstation running IRIS Showcase (v 3.3), or a Macintosh G4 workstation running Adobe Illustrator 8.0 and printed on a Fujix Pictrography color printer.

### RESULTS

### Subcellular Distribution of GK, GKRP and Glycogen Under Basal Conditions

Overnight-cultured primary hepatocytes maintained in 5.5mmol/l (basal) glucose were analyzed by immunocytochemistry and confocal imaging for GK and GKRP, and for PAS-reactive glycogen with a modified fluorescent method. Immunoblot analysis of a whole-cell homogenate of isolated hepatocytes was performed to determine the specificity of GK and GKRP antisera used in these studies, and this was confirmed by a single immunoreactive band for each (Fig. 1A). Under basal glucose conditions, both GK and GKRP were observed in the nucleus of hepatocytes (Figs. 1B,C). Higher magnification reveals an identical localization pattern of GK and GKRP within the nucleoplasm of hepatocytes upon DNA counterstaining (Fig. 1D). The fluorescent intensities of the respective nuclear GK and GKRP signals were generally similar, however, there was cell-to-cell variation in their absolute intensities attributable to isolation of primary hepatocytes with sustained heterogeneity with respect to metabolic zonation and glycolytic enzyme content.[23]

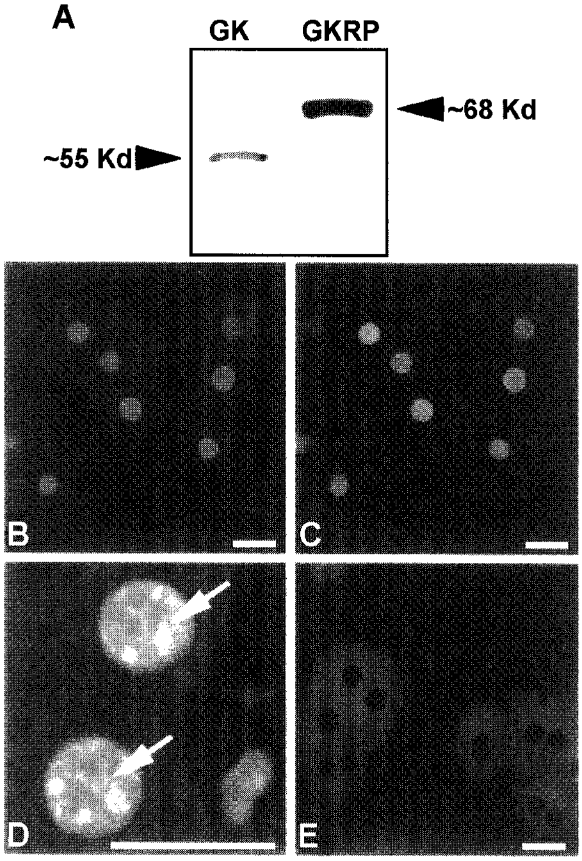

FIGURE 1 GK and GKRP immunoreactivity, and PAS-fluorescence in isolated hepatocytes cultured under basal glucose conditions. **A.** Representative immunoblot analysis of a whole-cell homogenate of freshly-isolated hepatocytes demonstrating monospecificity of GK and anti-GKRP antisera. The apparent molecular mass is indicated. **B.** Confocal immunofluorescence image of a representative field of hepatocytes immunostained for GK (red). The predominant staining signal is localized to the nuclei. **C.** Same field as in B immunostained for GKRP (green). Incubations of cells with pre-immune sera resulted in only low diffuse background staining (not shown). **D.** Hepatocytes *in situ* fluorescently stained for GK and GKRP with counterstained nuclei (green). Colocalization of GK (red) and GKRP (blue) in hepatocyte nuclei results in pink emission within the nucleoplasm, but it is clearly void from the heterochromatin and nucleoli (green). **E.** Fluorescent PAS technique reveals low levels of cytoplasmic PAS-reactive material. Hepatocytes subjected to basal glucose and stained with either the modified-fluorescent or the conventional PAS technique revealed no detectable staining when viewed by brightfield microscopy (not shown) (see Methods section for details). Scale bar represents 20 µm.

The established fluorescent method for demonstrating Schiff's-type polyaldehyde staining[24] yields a reaction product with a fluorescence emission peak that is unsuitable for optimal quantitative confocal analysis. For this reason, a modified PAS technique was devised and used for the fluorescence detection of newly synthesized glycogen. Control experiments for

PAS-staining included omission of either the oxidation or Schiff's-staining step and resulted in no detectable cytoplasmic fluorescence. Basal glucose-cultured hepatocytes stained with the modified PAS and visualized by laser confocal microscopy resulted in very low levels of residual PAS-reactive polysaccharide (Fig. 1E). These hepatocytes lacked detectable reactivity using conventional PAS staining (data not shown) suggesting that this baseline level of fluorescence corresponds to glycogen depletion under these basal glucose conditions.

### GK and GKRP Distribution and Glycogen Deposition Under Stimulating Conditions

Since both glucose and fructose are potent stimuli for GK release by GKRP in hepatocytes[25] we tested the effect of 0.2mmol/l fructose, in the presence of either basal (5.5mmol/l) or elevated but physiological (10mmol/l) glucose levels. Either or both of these substrates caused changes in both the subcellular localization of GK immunoreactivity and the relative levels of PAS-reactive glycogen (Fig. 2). Most hepatocytes exhibited a reduction in nuclear GK fluorescence with a corresponding increase in cytoplasmic signal, especially towards the cell surface opposing contacted cells (Fig. 2A). There were no substantial qualitative differences in the GK distribution patterns between hepatocytes subjected to the various substrate conditions, although differences in the magnitude of nuclear depletion were observed (see below). GKRP immunoreactivity remained confined to the nucleus under all stimulating conditions (Fig. 2B), in agreement with prior studies.[4,12] Confocal fluorescence imaging of PAS reactivity in 30min substrate-stimulated hepatocytes indicated that glycogen was initially deposited at the cell periphery (Fig. 2C). In hepatocytes stimulated for 60min, glycogen accumulation was maintained at the cell periphery but was also observed throughout the cytoplasm (Fig. 2D).

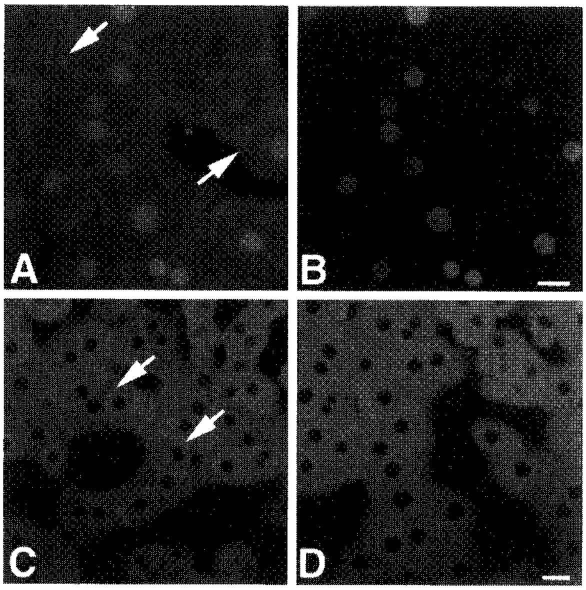

FIGURE 2 GK and GKRP immunoreactivity, and PAS-fluorescence in isolated hepatocytes cultured under substrate-stimulated conditions. **A.** Confocal immunofluorescence image of a representative field of hepatocytes immunostained for GK (red). Although hepatocytes retain variable levels of nuclear GK immunofluorescence, most exhibit increased cytoplasmic staining, especially at their surfaces (arrows). **B.** Same field as in B immunostained for GKRP (green). Note that GKRP immunofluorescence is confined to the nucleus. This pattern of GKRP staining was observed among all substrate stimulation conditions. **C.** Fluorescent PAS-staining of hepatocytes subjected to a 30min stimulation by 10mmol/l glucose showing a significant enhancement in cytoplasmic glycogen deposition with considerable accumulation at the cell surfaces (arrows). **D.** Hepatocytes stained for glycogen following a 60min stimulation by 10mmol/l glucose showing a further accumulation of glycogen signal distributed at the surface as well as throughout the cytoplasm. Scale bar equals 20$\mu$m.

### Semi-quantitative Analysis of GK and GKRP Distribution and Glycogen Deposition

To measure differences in the nucleocytoplasmic redistribution of GK and GKRP, and changes in glycogen deposition in stimulated hepatocytes, we assessed the ratio of nuclear to cytoplasmic (N:C) intensity from confocal images. Although the recorded cytoplasmic intensity values reflect only a portion of the total cytoplasmic area, we determined that recording fluorescence changes in an equivalent area of the cytoplasm as the nuclear surface area (~350 pixels) was an adequate representation of the trends throughout the entire cytoplasm as both perinuclear and peripheral cytoplasmic regions were sampled (see Methods section for details). Upon substrate stimulation there was a decrease in the GK N:C ratio (Fig. 3A). Hepatocytes incubated under basal glucose conditions exhibited a mean ratio for GK of 3.75 ± 0.12 (Fig. 3A). Hepatocytes incubated for 30min in 10mmol/l glucose, which approximates the post-absorptive plasma glucose concentration in the portal blood,[25] showed a significant (37%) reduction (2.36 ± 0.05) in this ratio. Fructose was found to strongly stimulate GK translocation both at basal and high glucose concentrations (Fig. 3A) (N:C ratio of 2.39 ± 0.07 and 2.48 ± 0.08, respectively), but in a manner similar to high glucose alone.

Substrate-induced GK translocation occurred within 30min, in agreement with a prior study.[4] In hepatocytes cultured for a total of 60min under stimulating conditions, cultures containing

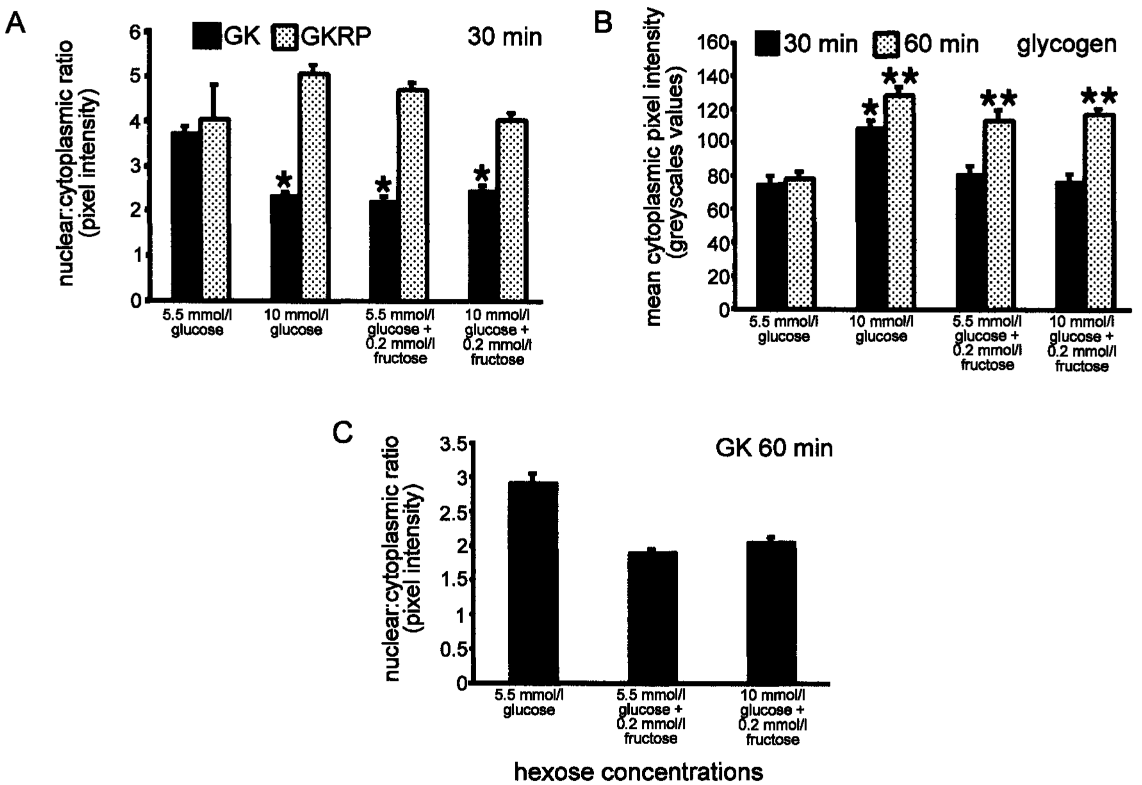

FIGURE 3 Substrate-stimulated GK translocation is associated with glycogen accumulation as determined by quantitative confocal fluorescence imaging. The relative distribution of GK immunoreactivity, GKRP immunoreactivity, and glycogen (PAS-reactive fluorescence) for each substrate condition and incubation period was expressed as either the mean N:C ratio (in pixel intensity) ± SEM (for GK and RP immunoreactivity) or as the mean cytoplasmic pixel intensity ± SEM (for glycogen). **A.** Subcellular distribution of GK (dark bars) and RP (stippled bars) expressed as changes in the average nuclear to cytoplasmic ratio (N:C ratio) after 30min stimulation. Values represent mean ratios ± SEM (n = 54 – 74 cells assayed for each group). The N:C ratio of GK fell by ~37% ($p < 3.44 \times 10^{-14}$) compared to basal glucose group, indicating translocation from the nucleus, whereas the N:C ratio of GKRP did not increase (mean = 4.51 ± 0.22), and actually rose in the high glucose and basal glucose + fructose groups. **B.** Glycogen accumulation following substrate stimulation as compared at 30min (dark bars) and 60min (stippled bars) as determined by changes in cytoplasmic pixel intensity (n = 25 – 92 cells assayed each group). At 30min stimulation, only the high glucose group exhibited a significant change in PAS fluorescence ($p < 3.2 \times 10^{-7}$), whereas after 60min stimulation, all groups displayed significantly increased PAS-reactive glycogen ($p < 1.9 \times 10^{-6}$). **C.** Subcellular distribution of GK expressed as N:C ratios after 60min stimulation (n = 58 – 78 cells). The 10mmol/l glucose-treated hepatocytes were not included due to high cellular autofluorescence observed after 60min exposure in three separate experiments. Both the basal glucose + fructose groups and high glucose + fructose groups showed significant decreases in the GK N:C ratio ($p < 2.03 \times 10^{-7}$ and $1.68 \times 10^{-6}$, respectively), whereas significant differences in the ratios of GKRP were not observed (not shown).

fructose with basal or high glucose exhibited even lower mean GK N:C ratios (1.92 ± 0.04 and 2.08 ± 0.06, respectively) (Fig. 3C). However, when compared to their respective control (2.94 ± 0.12), these values demonstrate a similar reduction of the GK N:C ratio as in the 30 min stimulation group. Therefore, substantial differences in the magnitude of GK translocation between 30 and 60 min of stimulation were not observed. Furthermore, analyses of GK fluorescence patterns in 60 min-high glucose alone-cultured hepatocytes resulted in disproportionately high nuclear and cytoplasmic intensities as the result of enhanced autofluorescence, therefore, those values were not included in Figure 3C. Similar patterns of high cellular autofluorescence were seen in 60 min-"high glucose only" cultured hepatocytes from two other isolations.

Potential subcellular changes in GKRP immunoreactivity were also determined in 30 min-stimulated hepatocytes. The relative intensity of the nuclear GKRP signal, when expressed as the mean N:C ratio index (4.04 ± 0.16 at basal glucose), actually showed an opposite relationship with hexose stimulation compared to that of GK, as it increased (average 4.60) under those conditions which maximally stimulate a reduction in the GK N:C (Fig. 3A). This is probably due to enhanced recognition of the protein by the GKRP antibody upon release of GK. The predominantly nuclear pattern of GKRP immunoreactivity was maintained in stimulated hepatocytes cultured for an additional 30 min (data not shown). This staining pattern is different from the subcellular GKRP patterns reported under supraphysiological glucose levels.[1,16]

Semi-quantitative analysis of glycogen accumulation, as determined by changes in mean cytoplasmic intensity, revealed maximal glycogen deposition in hepatocytes exposed to 10 mmol/l glucose alone (Fig. 3B). At 30 min, hepatocytes subjected to high glucose exhibited a 45% increase in PAS fluorescence over control hepatocytes. Interestingly, hepatocytes incubated in the presence of fructose with either basal or high glucose demonstrated no significant increase in PAS-generated fluorescent signal in this short timeframe (Fig. 3B). However, in hepatocytes stimulated for 60 min, there was significantly more PAS-reactive glycogen accumulated than was present at the 30 minute incubation (Fig. 3B). When compared to the basal glucose controls, 10 mmol/l glucose-cultured hepatocytes exhibited a 65% increase in fluorescence intensity while the fructose-treated cells supplemented with either basal or 10 mmol/l glucose showed an approximately 50% increase in PAS-reactivity. Thus it appears that in rat hepatocytes subjected to 10 mmol/l glucose alone there is significant stimulation of GK translocation within 30 min (Fig. 3A), and they are conducive to maximal glycogen deposition within both 30 and 60 min incubation periods (Fig. 3B).

### Subcellular Distribution of GK and Glycogen upon Substrate Stimulation

The distribution of GK and glycogen in basal and stimulated primary hepatocytes were determined by scanning in the "z" (depth) dimension using confocal microscopy and densitometry. In hepatocytes cultured in basal glucose for 30 min, XZ-plane confocal imaging and densitometric scanning confirm that the predominant GK immunofluorescence signal emanates from the nuclei, and not from a perinuclear site (Fig. 4A). In the presence of basal glucose, cultured hepatocytes subjected to the PAS fluorescence procedure and XZ-scanning demonstrated only a weak fluorescent signal associated with the hepatocyte cytoplasm and no signal in their nuclei (Fig. 4B). Upon substrate stimulation, XZ-plane imaging of hepatocytes exhibited decreased nuclear GK immunofluorescence and the appearance of peripherally-oriented GK (Fig. 5A). This compartmentalized GK signal is not entirely random at the cell surface but often shows a preference for the opposing face of adjacent hepatocytes. Importantly, surface-oriented glycogen deposition is observed in a pattern similar to that of GK upon substrate stimulation (Fig. 5B).

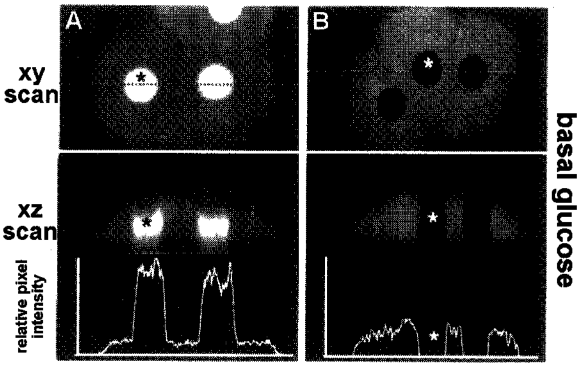

FIGURE 4 Subcellular distribution of GK and glycogen in unstimulated primary hepatocytes. Hepatocytes cultured in basal glucose 30min. **A**. (Top) XY-plane (conventional) confocal image of GK immunofluorescence showing strong nuclear signal. The asterisk marks a nucleus and the dashed line designates plane of optical section for reference in XZ scan below. Corresponding XZ-plane confocal image (middle) referenced in XY (conventional flat)-plane (dashed line). Densitogram of XY-plane image along dashed line in upper panel (bottom). Note predominant GK signal is associated with nuclei (* within peak in densitogram). **B**. Basal-glucose cultured hepatocytes subjected to PAS fluorescence procedure. Note that only a weak fluorescent signal emanates from cytoplasm. A nucleus is indicated (*) for reference.

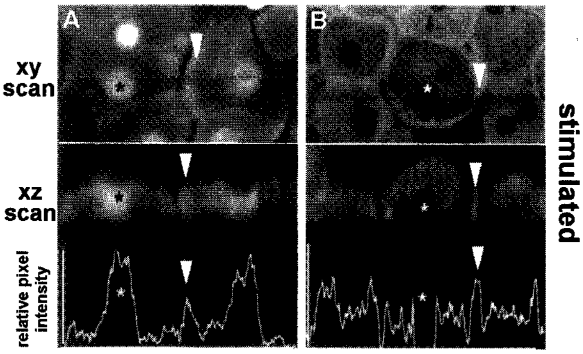

FIGURE 5 Subcellular distribution of GK and glycogen in stimulated primary hepatocytes. **A**. XY-plane image of hepatocytes incubated in 10mmol/l glucose+0.2mmol/l fructose for 60min showing slightly decreased nuclear GK signal but the appearance of surface-oriented GK (upper panel, arrowheads). Asterisk marks a nucleus and dashed line designates plane of section in XZ-plane (middle). Peripherally-sited GK signal is not random but is often concentrated at the opposing face of adjacent hepatocytes (arrowheads). **B**. Substrate-stimulated hepatocytes stained for glycogen. Surface-oriented glycogen deposition (upper panel, arrowheads) and a nucleus (*) are indicated for reference in XZ-scan (middle). Note similar patterns of surface localization (arrowheads) for GK and glycogen in stimulated hepatocytes.

### Glycogen Distribution in Perfused Liver

In order to validate the glycogen deposition patterns observed in cultured hepatocytes in a perfusion system with intact liver tissue, we performed conventional PAS staining in rat liver sections which were perfused with basal (5.5mmol/l) and high (20mmol/l) glucose. Liver subjected to basal glucose exhibited very low levels of PAS-reactivity (Fig. 6A), consistent with depleted glycogen stores in most hepatocytes after an overnight fast.

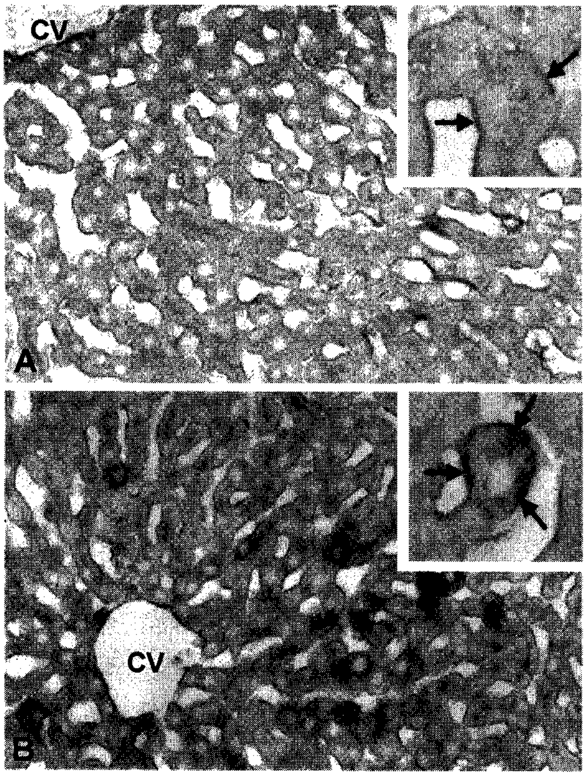

FIGURE 6 Glycogen accumulation in hepatocytes *in situ*. Perfused rat liver fixed, processed for paraffin embedding, and sections stained with the conventional PAS procedure. The central vein (cv) is indicated for reference. **A**. Overview of liver from a rat perfused for 30min with 5.5mmol/l glucose following an overnight fast. Note that hepatocytes are considerably depleted of PAS-reactive material. Inset shows higher magnification revealing that residual PAS-reactive material is non-glycogenic, but is attributable to basement membrane glycoproteins of the sinusoidal endothelium (arrows). **B**. Rat liver perfused for 30min with 20mmol/l glucose following a 30min basal glucose perfusion. Note that PAS-reactive glycogen is not uniformly synthesized among different hepatocytes. Higher magnification (inset) shows that glycogen is initially deposited at the periphery facing the sinusoidal space (arrows).

Twenty mmol/l glucose stimulated the deposition of PAS-reactive glycogen within a 30min timeframe (Fig. 6B). Glycogen is non-uniformly synthesized among different hepatocytes at this early stage of stimulation and is often localized towards the sinusoidal surface, a pattern consistent with the peripheral patterns observed in cultured hepatocytes under stimulating conditions.

### DISCUSSION

Hepatic GK has been shown to reversibly translocate between the nucleus and cytoplasm in response to specific hexose substrates, and this flux is coupled to glycogen synthesis by a biochemical process in which there is little morphological perspective. While the cellular mechanisms mediating GK translocation are beginning to be resolved, it is clear that under basal glucose conditions GK is bound to GKRP in the nucleus. We have performed studies directed at determining the impact of hexose substrate stimulation within the physiological range on the subcellular localization of GK and its spatial relationship with initial sites of glycogen deposition as determined by PAS-reactivity. Our results suggest that GK is released from nuclear-bound GKRP in response to increased substrate concentrations, that it translocates to a compartment within the peripheral cytoplasm close to the plasma membrane facilitating the channeling of glucose into glycogen.

### Subcellular Compartmentalization of GK and GKRP in Hepatocytes

It has previously been shown that under basal glucose conditions in a cell-free system, GK associates with the digitonin-stable matrix of the hepatocyte.[25] However, upon stimulation with glucose or small amounts of fructose, GK is liberated into the cytosolic compartment.[25] These data suggested that GKRP regulates GK by a reversible interaction that anchors the enzyme to the insoluble hepatocyte matrix.[3] The above observations were extended by immunohistochemical studies of hepatocytes demonstrating that GK was predominately located in the nucleus under basal glucose conditions[26] and that it co-distributes with GKRP.[1]

Although the functional relevance of the GK-GKRP interaction in the hepatocyte nucleus is well-accepted, there is some controversy regarding whether GKRP also translocates from the nucleus upon substrate stimulation and, hence, may play an active role in the GK translocation. Both GK and GKRP[27] were shown to translocate from the nucleus to cytoplasm upon 20 or 25mmol/l glucose stimulation in either isolated, perfused livers[1] or primary hepatocytes.[16] In contrast to these results, a study by a different group using primary hepatocytes subjected to more physiological, but high glucose levels (15.5mmol/l), demonstrated that while GK reversibly shuttles between the nucleus and cytoplasm upon substrate stimulation, GKRP remained localized within the nucleus.[4] Our studies, which have made use of both liver perfusion experiments (present study and unpublished observations by T. Jetton and M. Shiota) and in primary hepatocytes (present study), suggest that immunohistochemically demonstrable GKRP is retained within the nuclear compartment under several substrate stimulation conditions that mimic those of the portal blood following a meal.

### Alternative Roles for GKRP

Evidence is accumulating on additional roles for GKRP besides simply the regulation of GK catalytic activity. GK/GKRP cotransfection studies in non-hepatic cell lines by our group[17] and others[28] suggest that nuclear localization of GK and GKRP may be dependent on the presence of both proteins. We have recently shown that GKRP may not only function in the nuclear sequestration of GK, but may also regulate nuclear entry of GK by serving as a molecular chaperone.[17] For example, entry of GK into the nucleus appears to be dependent on binding and "piggy-backing" on GKRP, instead of the use of a specific GK nuclear localization sequence (NLS). Further, nuclear efflux of GK is probably the result of nuclear pore complex recognition of a specific leucine-rich nuclear export sequence,[17] but at present, it is unclear if this sequence operates in substrate-induced translocation in hepatocytes. Analyses of GKRP-deficient mice have demonstrated an altered stability of GK protein,[12,29] impaired glucose tolerance, and lowered glycogen synthesis.[12] In isolated hepatocytes from these GKRP knockout mice, and in heterologous non-hepatic cell lines transfected with GK alone,[17,28] GK is localized exclusively in the cytoplasm where it may be more vulnerable to degradation. Thus, a function for GKRP in the nuclear import of GK implicates an indirect stabilizing role of this protein role for GK. In the current study, we could not detect cytoplasmic GKRP in hepatocytes under any conditions. However, we cannot rule out the possibility that a small pool of GKRP, perhaps associated with the nuclear envelope, may have escaped detection. Our data suggest that although GKRP is unlikely to have a direct role in glycogen synthesis in the cytoplasm, it probably exerts its control over glycogen production by regulating the active, translocatable pool of GK.

### Functional Significance of GK Cytoplasmic Compartmentalization

There is mounting evidence that some key enzymes of carbohydrate metabolism, long considered to freely diffuse with their substrates throughout the cytoplasm may, in fact, be highly restricted in their subcellular location by transiently binding to the cytoplasmic matrix.[30,31] This binding may also involve flux between a bound and free state depending on substrate availability. The compartmentalization of certain glycolytic enzymes into organized assemblies may facilitate substrate channeling, whereby the product of one reaction is directed, now as a substrate, to the next enzyme in the assembly.[30–32] We have found that within hepatocytes subjected to substrate conditions mimicking portal blood in the post-absorptive state, GK immunoreactivity is situated at a peripheral site near the plasma membrane. However, we found that GK immunoreactivity is never localized on the surface in a pattern superimposable with GLUT2 immunoreactivity.[33] Glycogenic PAS-reactivity accumulates in glucose/fructose-stimulated hepatocytes in a pattern resembling that of cell surface-oriented GK, with the greatest signal initially occurring near the plasma membrane. Since this peripheral deposition of glycogen is also observed in the intact liver during substrate perfusion following a 24 fast, it is unlikely to be a cell culture artifact. As both GK[34] and glycogen synthase[15] have been previously localized to the actin-rich periphery of hepatocytes, the cortical cytoskeleton may provide a scaffold for these proteins for both the efficient channeling of glucose 6-phosphate between these enzymes in glycogenesis.

In contrast to its actions in the cytoplasm, it is not known whether GK plays any functional role in the nucleus. Although it remains to be proven, the available evidence indicates that GK, when bound to GKRP, is catalytically inactive. Toyoda *et al.*, surmise that the removal of GK from the cytoplasmic compartment may reduce the futile substrate cycle between glucose and G6P.[26,27] Brown *et al.*[4] have suggested that the binding of GK to GKRP in the nucleus may protect the enzyme from proteolysis and subsequent studies in GKRP knockout mice strongly imply that this is the case.[12,29] Thus, nuclear translocation of GK is probably a sequestration mechanism that restricts glucose phosphorylation until the appropriate nutritional/metabolic signals arise. On the other hand, one study reports the existence of nuclear-associated glycogen that co-distributes with several of its metabolizing/regulatory enzymes.[35] Interactions between glycogen and certain kinases may exist at the level of the nuclear envelope suggesting that a constitutive pool of glycogen may play a scaffolding and sequestration function for these enzymes.[35] Although we did not detect PAS-reactive glycogen in nucleoplasm of hepatocytes in the present study, we have observed glycogen aggregates at the nuclear-cytoplasmic boundary under stimulating conditions (T. Jetton and M. Shiota, unpublished data). More studies are warranted to determine if a residual pool of glycogen may have a role in GK nuclear sequestration or translocation.

### Physiological Significance of GK Translocation

Agius *et al.*[9] have shown that glycogen synthetic rates are maximal under those glucose concentrations (5–10mmol/l) which stimulate release of matrix-bound GK. Here we report that increased glycogen deposition occurs under the same stimulation conditions that induce maximal GK translocation from the nucleus (*i.e.*, 10mmol/l glucose). *In vivo* studies on the starved-to-fed metabolic changes in the rat liver have noted that glycogen begins to accumulate 1h following refeeding, and continues to increase at a fairly steady rate up to 24h.[36] In both primary cultured hepatocytes and liver tissue sections from fasted animals, glycogen staining, by the conventional or modified (fluorescent) PAS technique is virtually undetectable, consistent with the depletion of glycogen reserves. Here we have shown that glycógen accumulation can be cytochemically detected as early as 30min following substrate stimulation. Although fructose was found to be a potent stimulator of GK translocation both at basal and high glucose concentrations, within the 60min time course of the present study, unexpectedly, the 10mmol/l glucose-treated hepatocytes exhibited the greatest glycogen accumulation. In part, this may be explained by enhanced activity of GK at 10mmol/l glucose resulting in a proportional increase in the substrate glucose 6-phosphate (G6P) leading to accelerated rates of glycogen synthesis compared to the basal glucose/fructose-treated group. It is noteworthy that the high glucose plus fructose-treated hepatocytes failed to demonstrate an additive effect on glycogen deposition.

The increased cytoplasmic GK immunofluorescence observed at the cell periphery was similar to the pattern observed for newly deposited glycogen suggesting that the peripherally-compartmentalized GK directly facilitates glycogen synthesis. While all cytoplasmic GK is thought to be kinetically active, surface-oriented GK would logically favor efficient glucose phosphorylation at a localized site for rapid glucose disposal into glycogen. This may involve the activation and parallel translocation of glycogen synthase[15] by GK-generated G6P.[10] The physiological relevance and potential interrelationships between glucose, fructose, sorbitol, various sugar phosphates, and intermediates of glycolysis in the modulation of GK activity through GKRP is unclear. Nonetheless, it is becoming increasingly evident from analyses of GK overexpression models both *in vitro*[37] and *in vivo*[22] that hepatic GK leverages a much tighter control over glucose homeostasis than may have been previously thought. Thus, short-term mechanisms for modulation of GK activity, although likely to be complex, have probably evolved to insure that a range of absorbed nutrients in the portal blood fine tune the rate of glucose metabolism in the hepatocyte.

### CONCLUSIONS

Our results suggest that GKRP, a nuclear anchoring protein that sequesters and allosterically inhibits GK within the hepatocyte nucleus under basal glucose conditions, upon substrate stimulation, releases GK proportionally according to the concentration and nature of the specific substrate. Once released, GK rapidly translocates from the nucleus and becomes compartmentalized at the cell periphery where glycogen is initially deposited promoting efficient glucose channeling into the glycogen synthetic pathway.

### *Acknowledgments*

We thank Dr. J. F. Grippo, Department of Metabolic Diseases, Hoffman-LaRoche, Inc., for his gift of the rabbit anti-GKRP antiserum.

These studies were supported both by a Pilot and Feasibility Award (to TLJ) from the Vanderbilt Diabetes Research and Training Center (DK20593), and by grants (to MAM) from the NIH to MAM (DK42612 and DK 42502) and DWP (DK53434). Confocal images were acquired and analyses performed using the Cell Imaging Shared Resource (supported by CA68485 and DK20593).

### References

[1] Toyoda, Y., Miwa, I., Satake, S., Anai, M. and Oko, Y. (1995a). Nuclear localization of the regulatory protein of glucokinase in rat liver and translocation of the regulator to the cytoplasm in response to high glucose. *Biochem. Biophys. Res. Comm.*, **215**, 467–473.

[2] Van Schaftingen, E. (1994). Short-term regulation of glucokinase. *Diabetologia*, **37**, S43–S47.

[3] Agius, L., Peak, M. and Schaftingen, E. V. (1995). The regulatory protein binds to the hepatocyte matrix, but, unlike glucokinase, does not translocate during substrate stimulation. *Biochem. J.*, **309**, 711–713.

[4] Brown, K. S., Kalinowski, S. S., Megill, J. R., Durham, S. K. and Mookhtiar, K. A. (1997). Glucokinase regulatory protein may interact with glucokinase in the hepatocyte nucleus. *Diabetes*, **46**, 179–186.

[5] Fernandez-Novell, J. M., Castel, S., Bellido, D., Ferrer, J. C., Vilaro, S. and Guinovart, J. J. (1999). Intracellular distribution of hepatic glucokinase and glucokinase regulatory protein during the fasted to refed transition in rats. *FEBS Lett.*, **459**, 211–214.

[6] Toyoda, Y., Miwa, I., Kamiya, M., Ogiso, S., Nonogaki, T., Aoki, S. and Okuda, J. (1995b). Tissue and subcellular distribution of glucokinase in rat liver and their changes during fasting-refeeding. *Histochemistry*, **103**, 31–38.

[7] Vandercammen, A. and Van Schaftingen, E. (1993). Species and tissue distribution of the regulatory protein of glucokinase. *Biochem. J.*, **294**, 551–556.

[8] Toyoda, Y., Ito, Y., Tanigawa, K. and Miwa, I. (2000). Impairment of glucokinase translocation in cultured hepatocytes from OLETF and GK rats, animal models of type 2 diabetes. *Arch. Histol. Cytol.*, **63**, 243–248.

[9] Agius, L., Peak, M., Newgard, C. B., Gomex-Foix, A. M. and Guinovart, J. J. (1996). Evidence for a role of glucose-induced translocation of glucokinase in the control of hepatic glycogen synthesis. *J. Biol. Chem.*, **271**, 30479–30486.

[10] Seoane, J., Gomez-Foix, A. M., O'Doherty, R. M., Gomez-Ara, C., Newgard, C. B. and Guinovart, J. J. (1996). Glucose-6-phosphate produced by glucokinase, but not hexokinase I, promotes the activation of hepatic glycogen synthase. *J. Biol. Chem.*, **271**, 23756–23760.

[11] Postic, C., Shiota, M., Niswender, K. D., Jetton, T. L., Chen, Y., Moates, J. M., Shelton, K. D., Lindner, J., Cherrington, A. D. and Magnuson, M. A. (1999). Dual roles for glucokinase in glucose homeostasis as determined by liver and pancreatic beta cell-specific gene knock-outs using Cre recombinase. *J. Biol. Chem.*, **274**, 305–315.

[12] Farrelly, D., Brown, K. S., Tieman, A., Ren, J., Lira, S. A., Hagan, D., Gregg, R., Mookhtiar, K. A. and Hariharan, N. (1999). Mice mutant for glucokinase regulatory protein exhibit decreased liver glucokinase: a sequestration mechanism in metabolic regulation. *Proc. Natl. Acad. Sci.* USA, **96**, 14511 – 14516.

[13] de la Iglesia, N., Mukhtar, M., Seoane, J., Guinovart, J. J. and Agius, L. (2000). The role of the regulatory protein of glucokinase in the glucose sensory mechanism of the hepatocyte. *J. Biol. Chem.*, **275**, 10597–10603.

[14] Villar-Palasi, C. and Guinovart, J. J. (1997). The role of glucose-6-phosphate in the control of glycogen synthase. *FASEB J.*, **11**, 544–558.

[15] Fernandez-Novell, J. M., Bellido, D., Vilaro, S. and Guinovart, J. J. (1997). Glucose induces the translocation of glycogen synthase to the cell cortex in rat hepatocytes. *Biochem. J.*, **321**, 227–231.

[16] Mukhtar, M., Stubbs, M. and Agius, L. (1999). Evidence for glucose and sorbitol-induced nuclear export of glucokinase regulatory protein in hepatocytes. *FEBS Lett.*, **462**, 453–458.

[17] Shiota, C., Coffey, J., Grimsby, J., Grippo, J. F. and Magnuson, M. A. (1999). Nuclear import of hepatic glucokinase depends on the glucokinase regulatory protein, whereas nuclear export is due to a nuclear export signal sequence in glucokinase. *J. Biol. Chem.*, **274**, 37125–37130.

[18] Shiota, M., Inagami, M., Fujimoto, Y., Moriyama, M., Kimura, K. and Sugano, T. (1995). Cold acclimation induces zonal heterogeneity in gluconeogenic responses to glucagon in rat liver lobule. *Am. J. Physiol.*, **268**, E1184–E1191.

[19] Liang, Y., Jetton, T. L., Zimmerman, E., Najafi, H., Matschinsky, F. M. and Magnuson, M. A. (1991). Effects of alternate RNA splicing on glucokinase isoform activities in the pancreatic islet, liver, and pituitary. *J. Biol. Chem.*, **266**, 6999–7007.

[20] Kaur, S., Jetton, T., Kurylko, G., Hope, W., Lucas, D. A., Bhatt, H., Coffey, J. W., Lusch, L., Flint, N., Yagaloff, K. A., Magnuson, M. and Grippo, J. F. (1996). Cloning, expression, and characterization of mouse liver glucokinase regulatory protein. *Proceedings of the 1997 Keystone Symposia "Insulin action and secretion in NIDDM"* (Abstract).

[21] Jetton, T. L., Liang, Y., Pettepher, C. C., Zimmerman, E. C., Cox, F. G., Horvath, K., Matchinsky, F. M. and Magnuson, M. A. (1994). Analysis of uptream glucokinase promoter activity in transgenic mice and identification of glucokinase in rare neuroendocrine cells in the brain and gut. *J. Biol. Chem.*, **269**, 3641–3654.

[22] Niswender, K. D., Postic, C., Jetton, T. L., Bennett, B. D., Piston, D. W., Efrat, S. and Magnuson, M. A. (1997). Cell-specific expression and regulation of a glucokinase gene locus transgene. *J. Biol. Chem.*, **272**, 22564–22569.

[23] Jungermann, K. and Keitzmann, T. (1996). Zonation of parenchymal and non-parenchymal metabolism in liver. *Ann. Rev. Nutr.*, **16**, 179–203.

[24] Weinblatt, F. M., Shannon, W. A. and Seligman, A. M. (1975). A new fluorescent method for the demonstration of macromolecular aldehydes. *Histochemistry*, **41**, 353–359.

[25] Agius, L. and Peak, M. (1993). Intracellular binding of glucokinase in hepatocytes and translocation by glucose, fructose, and insulin. *Biochem. J.*, **296**, 785–796.

[26] Miwa, I., Mitsuyama, S., Toyoda, Y., Nonogaki, T., Aoki, S. and Okuda, J. (1990). Evidence for the presence of rat liver glucokinase in the nucleus as well as in the cytoplasm. *Biochem. Int.*, **22**, 759–767.

[27] Toyoda, Y., Miwa, I., Kamiya, M., Ogiso, S., Nonogaki, T., Aoki, S. and Okuda, J. (1994). Evidence for glucokinase translocation by glucose in rat hepatocytes. *Biochem. Biophys. Res. Comm.*, **204**, 252–256.

[28] Bosco, D., Meda, P. and Iynedjian, P. B. (2000). Glucokinase and glucokinase regulatory protein: mutual dependence for nuclear localization. *Biochem. J.*, **348**, 215–222.

[29] Grimsby, J., Coffey, J. W., Dvorozniak, M. T., Magram, J., Li, G., Matschinsky, F. M., Shiota, C., Kaur, S., Magnuson, M. A. and Grippo, J. F. (2000). Characterization of glucokinase regulatory protein-deficient mice. *J. Biol. Chem.*, **275**, 7826–7831.

[30] Al-Habori, M. (1995). Microcompartmentation, metabolic channeling and carbohydrate metabolism. *Int. J. Biochem. Cell Biol.*, **27**, 123–132.

[31] Knull, H. R. and Walsh, J. L. (1992). Association of glycolytic enzymes with the cytoskeleton. *Curr. Top. Cell Reg.*, **33**, 15–30.

[32] Welch, G. R. and Easterby, J. S. (1994). Metabolic channeling *versus* free diffusion: transition-time analysis. *Trends Biochem. Sci.*, **19**, 103–107.

[33] Thorens, B., Sarkar, B., Kaback, H. K. and Lodish, H. F. (1988). Cloning and functional expression in bacteria of a novel glucose transporter present in liver, intestine, kidney, and beta-pancreatic islet cells. *Cell*, **55**, 281–291.

[34] Murata, T., Katagiri, H., Ishihara, H., Shibasaki, Y., Asano, T., Toyoda, Y., Pekiner, B., Pekiner, C., Miwa, I. and Oka, Y. (1997). Co-localization of glucokinase with actin filaments. *FEBS Lett.*, **406**, 109–113.

[35] Ragano-Caracciolo, M., Berlin, W. K., Miller, M. W. and Hanover, A. A. (1988). Nuclear glycogen and glycogen synthase kinase 3. *Biochem. Biophys. Res. Comm.*, **249**, 422–427.

[36] Holness, M. J., MacLennan, P. A., Palmer, T. N. and Sudgen, M. C. (1988). The disposition of carbohydrate between glycogenesis, lipogenesis, and oxidation in liver during the starved-to-fed transition. *Biochem. J.*, **252**, 325–330.

[37] O'Doherty, R. M., Lehman, D. L., Seoane, J., Gomez-Foix, A. M., Guinovart, J. J. and Newgard, C. B. (1996). Differential metabolic effects of adenovirus-mediated glucokinase and hexokinase I overexpression in rat primary hepatocytes. *J. Biol. Chem.*, **271**, 20524–20530.

